# Correlation between benefit finding and caregiving abilities among family caregivers of patients with lung cancer: a network analysis

**DOI:** 10.3389/fpsyg.2024.1403919

**Published:** 2024-11-20

**Authors:** Xiaoyuan Lin, Haidan Xie, Ziqing Chen, Qi Zhao, Xiaozhou Zhou

**Affiliations:** ^1^Department of Nursing, The First Affiliated Hospital of Guangdong Pharmaceutical University, Guangzhou, Guangdong, China; ^2^School of Nursing, Guangdong Pharmaceutical University, Guangzhou, Guangdong, China

**Keywords:** lung cancer care quality, family caregiver, benefit finding, caregiving abilities, network analysis

## Abstract

**Objective:**

This study aimed to construct a network analysis model for benefit finding (BF) and caregiving abilities to clarify the interrelationships and mutual influences among different dimensions and propose nursing strategies.

**Methods:**

Convenience sampling was used to select 272 family caregivers of patients with lung cancer admitted to a tertiary hospital in Guangzhou City, China, from April 2023 to November 2023. Socio-demographic and disease characteristics questionnaire, the revised BF Scale, and the Family Caregiver Task Inventory (FCTI) were used for data collection. The R software was used to construct networks for BF and caregiving abilities, and network analysis methods were employed to identify network characteristics, core nodes, and bridge nodes.

**Results:**

In the overall network, the social relationship dimension (rs = 3.04) exhibited the highest strength centrality index, followed by the family relationship dimension (rs = 2.94). In addition, the social relationship dimension (rbs = 0.30) had the highest bridge strength centrality index, followed by the dimension of addressing personal emotional needs (rbs = 0.26).

**Conclusion:**

This study provides a new perspective on exploring the underlying mechanisms of interaction among different dimensions of BF and caregiving abilities in family caregivers of patients with lung cancer using network analysis. The findings suggest that healthcare professionals can improve family caregivers’ social relationships, family relationships and address emotion regulation to enhance BF and caregiving abilities. Specific nursing strategies are proposed, offering new intervention targets for enhancing BF and caregiving abilities among family caregivers of patients with lung cancer.

## 1 Introduction

Lung cancer, being one of the most prevalent malignant tumors, poses a significant global threat to human life and health due to its high incidence, mortality, and low cure rates (Xia et al., 2022). The diagnosis of lung cancer represents a major stressful event for both the patient and the entire family, as it entails a prolonged treatment journey. Alongside the treatment, the patient experiences symptoms such as nausea, fatigue, pain, and overall exhaustion, leading to an increased demand for caregiving (Filchner et al., 2022). Family caregivers play a crucial role in the caregiving system for lung cancer patients. Family caregivers refer to individuals, such as patient’s spouses, children, siblings, and other family members, who provide unpaid care services to family members with physical, psychological, cognitive, and other impairments (Sun et al., 2019).

Family caregivers are responsible for various tasks, including acquiring treatment information, participating in treatment decision-making, managing medical expenses, providing daily care and participating in disease management for the patient (Gao et al., 2020). The caregiving capacity of caregivers serves as a significant criterion for evaluating their ability to meet patients ‘physical and psychosocial needs, seek social support, and effectively carry out caregiving tasks (Kosberg and Cairl, 1986). However, studies have shown (Reitzel et al., 2022; Rassouli et al., 2023) that family caregivers often exhibit lower levels of caregiving capacity due to a lack of systematic knowledge training and skill guidance. They have shortcomings in observing the patient’s condition and lack effective coping strategies to overcome obstacles encountered during the caregiving process. As a result, family caregivers frequently experience negative emotions (Fleitas Alfonzo et al., 2022). Excessive attention to negative emotions may deepen caregivers’ cognitive awareness of their emotions and result in secondary harm. In recent years, researchers have turned their attention to the fact that caregivers can develop positive psychological traits even under the burden of heavy caregiving responsibilities and the influence of negative emotions.

Benefit finding, an important component of positive psychology, refers to the cognitive adaptation and adjustment of behavioral response to manage stressful events and derive positive meaning from them (Affleck and Tennen, 1996; Linley and Joseph, 2004). Several studies (Cheng et al., 2022; Cheng et al., 2017) have indicated that caregivers’ BF from the illness can alleviate their negative emotional experiences. At the same time, a positive psychological state can enhance caregivers’ competence, reducing the caregiving burden. Yan et al. (2023) conducted semi-structured interviews with caregivers of colorectal cancer patients and found that the improvement in caregiving competence was a significant aspect of caregivers’ personal growth, allowing them to perceive the benefits from the caregiving process. Research has shown (Yue et al., 2022) that there is a negative correlation between benefit finding and caregiving competence in caregivers of stroke patients; that is, the higher the caregiving competence (the lower the caregiving competence score), the higher the level of benefit finding.

Accurately explaining and elucidating the relationship between human psychological activities and behavioral changes is one of the important goals of clinical nursing psychology (Yuqing et al., 2020). Benefit finding, as a positive experience of caregivers in the caregiving process, has diverse sources of perceived benefits at personal, familial, and societal levels. Similarly, caregiving abilities, as an objective reflection of caregivers’ caregiving behavioral capacity, also involve improvements in various aspects of individual abilities. Therefore, it is crucial to clarify the underlying mechanisms within each dimension. Specifically, most studies (Yue et al., 2022) only explore the relationship between two variables using simple linear relationships, based on overall scores. When there are many variables, spurious correlations are likely to occur. This is when two originally unrelated variables show statistical differences without controlling for other variables, leading to biased conclusions (He et al., 2022). Additionally, the associations between the different dimensions of caregiving abilities and benefit finding, as well as the interplay between these dimensions, have not been clearly elucidated, resulting in less targeted and less effective clinical interventions.

Network analysis (Yuqing et al., 2020) can evaluate the relationship between any two nodes while controlling for their connections to other nodes, and identify central and bridging nodes as effective targets for clinical interventions. When interventions are applied to core nodes and bridge nodes, not only can the levels and effectiveness of the nodes themselves be improved, but the closely connected nodes can also be enhanced, thereby activating the entire network and achieving a ripple effect. This approach can greatly enhance the effectiveness of clinical interventions (Castro et al., 2019).

## 2 Method

### 2.1 Study design and participants

A cross-sectional study was conducted from April to November 2023, using convenience sampling to recruit family caregivers of patients with lung cancer who were receiving treatment at a tertiary hospital’s oncology and thoracic surgery departments in Guangzhou, China. Inclusion criteria were as follows: Patients: diagnosed with lung cancer through pathological or cytological examination. Family caregivers: (Xia et al., 2022) family members of the patients, including spouses, parents, children, in -laws, etc. (Filchner et al., 2022) primarily responsible for caregiving during the patient’s hospitalization and after discharge, with caregiving hours ≥8 h per day (Sun et al., 2019) aged ≥18 years, conscious, and able to read and understand the questionnaire items. Exclusion criteria were as follows: Patients: (Xia et al., 2022) severe cognitive impairment, severe sensory or language impairments; (Filchner et al., 2022) history of mental illness or psychiatric disorders, impaired consciousness. Family caregivers (Xia et al., 2022) suffering from severe chronic diseases, such as malignant tumors, heart or kidney failure, or respiratory failure (Filchner et al., 2022) receiving caregiving remuneration. The study has obtained ethical approval from the Ethics Committee of the First Affiliated Hospital of Guangdong Pharmaceutical University (Approval No.: MedEthics2023 No.13).

According to the estimation method for sample size in network analysis (Epskamp et al., 2018), at least P(P-1)/2 parameters are required when there are P nodes in the network. Following the standard of having at least 3–5 individuals per parameter to ensure the statistical power of the network analysis, a minimum sample size of 135–225 individuals would be needed. In the network analysis of this study, there are 10 dimensions in the BF scale and FCTI, with each dimension representing one node. This requires the estimation of 45 parameters (10*9/2). With a final sample size of 272 participants included in this study, it is sufficient to conduct the network analysis.

### 2.2 Research instruments

Sociodemographic and disease characteristics questionnaire: The questionnaire comprises two sections, one for patients and the other for caregivers. The socio-demographic data for patients and caregivers encompass age, gender, and educational level. Disease-related information encompasses whether the patient is undergoing primary treatment, the duration of disease diagnosis (in months), clinical stage, presence of metastasis, and treatment modalities. Caregiver-related information includes the relationship with the patient, duration of accumulated caregiving (in months), and the presence of other co-caregivers.

Revised version of the Benefit Finding Scale (BFS): The Chinese version of the Benefit Finding Scale, modified by Bian et al. (2018), was employed in this research. It comprises 22 items grouped into 5 dimensions: Acceptance (items 1–3), Family Relationships (items 4–9), Personal Growth (items 10–16), Social Relationships (items 17–19), and Health Behavior (items 20–22). Participants rate their level of BF on a 5-point Likert scale ranging from “completely absent” to “very much.” The Cronbach’s alpha coefficient for this scale in the present study was 0.938.

Family Caregiver Task Inventory (FCTI): The Family Caregiver Task Inventory, originally developed by Clark and Rakowski (1983), was utilized in this study using the Chinese version translated and revised by Lee and Mok (2011). This inventory primarily evaluates caregivers’ perceived difficulty level in undertaking caregiving tasks. It comprises 25 items, with a total score ranging from 0 to 50. Scores between 0 and 2 are assigned to reflect the degree of difficulty, ranging from “not difficult” to “extremely difficult.” Higher scores indicate more incredible difficulty in caregiving tasks and lower caregiving abilities. The Cronbach’s alpha coefficient for this inventory in the present study was 0.868.

### 2.3 Data collection

A questionnaire survey method was employed, and data collection was conducted by two trained researchers who followed standardized procedures. The researchers comprehensively explained the study’s purpose, process, and significance to the eligible participants. After obtaining informed consent from the patients and their caregivers, the questionnaires were distributed to them. The patients and caregivers self-reported sociodemographic information in the general information survey based on their circumstances, while disease-related information was cross-checked and completed by the two investigators through a review of the hospital medical records system. If the patients or caregivers had any difficulties or uncertainties regarding the questionnaire items, the researchers objectively clarified and addressed their concerns. After completion, the researchers conducted on-site verification and inspection of the questionnaires. If any incomplete items were identified, the participants were promptly informed to provide the missing information. A total of 296 questionnaires were distributed in this study, and 24 invalid questionnaires were excluded due to missing entries and other reasons. Ultimately, 272 valid questionnaires were obtained, resulting in a questionnaire response rate of 91.9%.

### 2.4 Data analysis

Data analysis was performed using R software version 4.3.2. Descriptive statistics were reported as (mean ± standard deviation) for normally distributed data and M (interquartile range) for skewed data. The network model was established using the “qgraph” package in R software, and the Spearman correlation analysis with the “EBICglasso” function was used to examine the relationship between benefit finding and caregiving abilities. This analysis aimed to determine an optimal network. In the network model of this study, each dimension in the BF and FCTI was treated as a “node,” and the correlations between each dimension were considered as “edges.” The thickness of the edges reflected the strength of the associations between the nodes, where thicker edges indicated stronger relationships. In network analysis (Castro et al., 2019), nodes with high centrality are considered core nodes as they interact with most other nodes in the network and can activate other nodes, thereby improving the overall network. Bridge nodes, on the other hand, are key nodes that connect relationships between different variables. Centrality in the network is commonly evaluated using three indicators: strength centrality, closeness centrality, and betweenness centrality. Higher values indicate greater importance in the network. Previous studies (Hallquist et al., 2021) have shown that strength centrality (the sum of absolute edge weights between a node and all directly connected nodes) is the most stable indicator. When there is a discrepancy in the ranking of these centrality measures, the ranking based on strength centrality is generally considered. Therefore, we used the strength centrality measure (rs) to identify core nodes in this study. Additionally, to assess the connectivity between the benefit finding and caregiver task inventory, the “network tools” package in R software was used, and the bridge strength centrality measure (rbs) was used to identify bridge nodes.

## 3 Results

### 3.1 Sociodemographic and disease characteristics of lung cancer patients and their caregivers

Among the lung cancer patients, there were more males, with 186 males (68.4%) and 86 females (31.6%). The age ranged from 28 to 90 years, with an average age of (59.29 ± 11.14) years. Most lung cancer patients had been diagnosed either for less than 3 months or for more than 1 year, with most patients being in the middle to late stages: 72 patients (26.5%) in stage III and 108 patients (39.7%) in stage IV. The current treatment methods primarily involve combinations of two or more treatments, including surgery combined with chemotherapy, immunotherapy combined with chemotherapy, or in combination with targeted therapy, radiation therapy, etc.

Among the caregivers, there were slightly more females (147, 54%) compared to males (125, 46%). The age of caregivers ranged from 19 to 77 years, with an average age of (44.4 ± 12.84) years. The majority of caregivers were either spouses or children of the patients, with 122 (44.9%) being spouses and 112 (41.2%) being children. The duration of caregiving was predominantly either less than 3 months or 1 year or more, with 122 (44.9%) providing care for less than 3 months and 92 (33.8%) for 1 year or more (Table 1).

**Table 1:** Sociodemographic and disease characteristics of patient and their caregiver.

Variables	*n* (%)	Variables	*n* (%)
Patient		Caregiver	
Gender		Gender	
Male	186 (68.4)	Male	125 (46.0)
Female	86 (31.6)	Female	147 (54.0)
Age		Age	
<40	16 (5.9)	<40	104 (38.2)
40–59	123 (45.2)	40–59	129 (47.4)
≥60	133 (48.9)	≥60	39 (14.3)
Duration since diagnosis (in months)		Employment status	
<12	173 (63.6)	Employed	147 (54.0)
12~	99 (36.4)	Retired	60 (22.1)
Clinical stage		Unemployed	65 (23.9)
I	46 (16.9)	Education level	
II	46 (16.9)	Under Elementary school	33 (12.1)
III	72 (26.5)	Junior high school	90 (33.1)
IV	108 (39.7)	High school	66 (24.3)
First-time treatment		College or above	83 (30.5)
Yes	67 (24.6)	Household income (in months)	
No	205 (75.4)	<2000	44 (16.2)
Metastasis		2000–3,999	58 (21.3)
Yes	210 (77.2)	4,000–5,999	85 (31.3)
No	62 (22.8)	≥6,000	85 (31.3)
Treatment method		Relationship with patient	
1	100 (36.8)	Spouse	122 (44.9)
2	106 (39.0)	Others	150 (55.1)
≥ 2	66 (24.2)	Duration of care (in months)	
Self-care level		<12	180 (66.2)
Fully independent	137 (50.3)	≥12	92 (33.8)
Partially independent	109 (40.1)	Co-caregivers	
Unable to care for self	26 (9.6)	No	96 (35.3)
		Yes	176 (64.7)

### 3.2 BF scores and dimension scores

The total BF score for caregivers of lung cancer patients was (73.10 ± 19.30), with a minimum of 22 and a maximum of 110. Among the dimensions, the highest score was for the family relationships dimension (3.54 ± 0.89), and the lowest was for the acceptance dimension (2.98 ± 1.02) (Table 2).

**Table 2:** BF scores and dimension scores.

Items	Number of entries	Min	Max	M	SD
Sum score of BF	22	22	110	73.10	19.30
Acceptance	3	1	15	8.94	3.05
Family relationships	6	6	30	21.24	5.31
Personal growth	7	7	35	23.96	6.86
Social relationships	3	3	15	9.55	3.42
Health Behavior	3	3	15	9.41	3.58

### 3.3 FCTI score and dimension scores

The FCTI score for caregivers of lung cancer patients was 10 (interquartile range 11.5), with a minimum of 0 and a maximum of 50 (see Table 3).

**Table 3:** FCTI scores and dimension scores.

Items	Number of entries	Min	Max	M	IQR
Sum score of FCTI	25	0	50	10	15.75
Adapting to caregiving roles	5	0	10	2	4
Coping and providing assistance	5	0	10	1	2
Managing personal emotions	5	0	10	2	4
Assessing family and community resources	5	0	10	2	3
Adjusting personal needs	5	0	10	1	3

### 3.4 Network model

**Figure 1:**
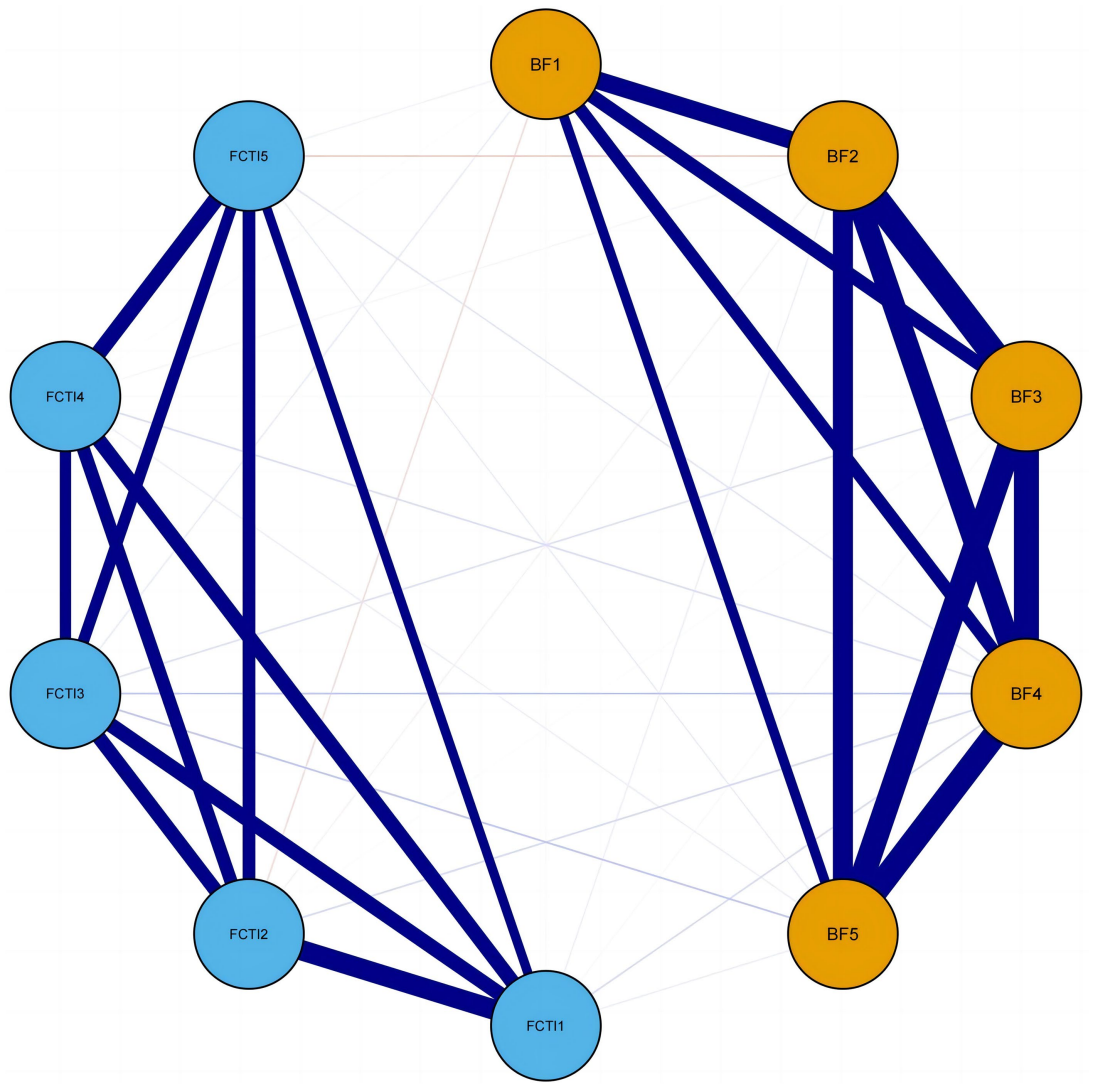
Network model of BF and FCTI among family caregivers of patients with lung cancer. BF1, acceptance dimension; BF2, family relationships dimension; BF3, personal growth dimension; BF4, social relationships dimension; BF5, health behaviors dimension; FCTI1, adaptation to caregiving roles dimension; FCTI2, coping and providing assistance dimension; FCTI3, managing personal emotions dimension; FCTI4, assessing family and community resources dimension; FCTI5, adjusting personal needs dimension.

Figure 1 illustrates the network model of BF and caregiving abilities among family caregivers of patients with lung cancer. The BF dimensions are represented in orange, while the FCTI dimensions are represented in blue. Solid blue lines indicate positive correlations and solid red lines indicate negative correlations. The accuracy test of edge weights demonstrated a narrow 95% confidence interval, indicating good network precision.

Table 4 displays the specific values of the edge weights in the network. All five dimensions of BF exhibit positive correlations. The strongest connection is observed between family relationships (BF dimension 2) and personal growth (BF dimension 3) with a weight of *r* = 0.77, followed by personal growth (BF dimension 3) and social relationships (BF dimension 4) with a weight of *r* = 0.74. Similarly, all five dimensions of caregiving abilities show positive correlations. The strongest connection is observed between adaptation to caregiving roles (FCTI dimension 1) and coping and providing assistance (FCTI dimension 2) with a weight of r = 0.67, followed by assessing family and community resources (FCTI dimension 4) and adjusting personal needs (FCTI dimension 5) with a weight of *r* = 0.49.

**Table 4:** Edge weights of the network linking BF and FCTI among family caregivers of patients with lung cancer.

	BF1	BF2	BF3	BF4	BF5	FCTI1	FCTI2	FCTI3	FCTI4	FCTI5
BF1	0.00									
BF2	0.64	0.00								
BF3	0.57	0.77	0.00							
BF4	0.56	0.70	0.74	0.00						
BF5	0.52	0.67	0.73	0.73	0.00					
FCTI1	−0.02	0.03	0.02	0.06	0.03	0.00				
FCTI2	−0.05	−0.03	−0.01	0.06	0.00	0.67	0.00			
FCTI3	0.03	0.00	0.05	0.09	0.09	0.59	0.58	0.00		
FCTI4	0.01	0.02	0.01	0.05	0.03	0.59	0.57	0.54	0.00	
FCTI5	0.02	−0.09	0.00	0.04	0.03	0.51	0.56	0.54	0.61	0.00

Regarding the inter-group connections, the weights of these connections are weaker compared to the intra-group connections. Negative correlations are observed between acceptance (BF dimension 1) and adaptation to caregiving roles (FCTI dimension 1), acceptance (BF dimension 1) and coping and providing assistance (FCTI dimension 2), family relationships (BF dimension 2) and coping and providing assistance (FCTI dimension 2), personal growth (BF dimension 3) and coping and providing assistance (FCTI dimension 2), and family relationships (BF dimension 2) and adjusting personal needs (FCTI dimension 5) with weights of *r* = −0.02, *r* = −0.05, *r* = −0.03, *r* = −0.01, and *r* = −0.09, respectively. The remaining inter-group connections exhibit positive correlations.

### 3.5 Node centrality index and identification of core nodes

**Figure 2:**
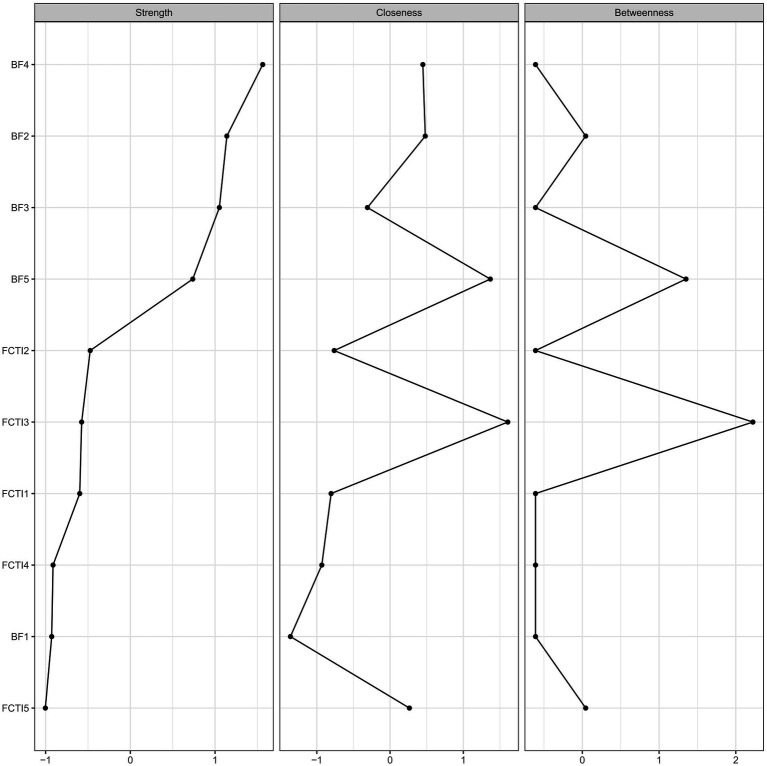
Centrality index of network nodes.

Figure 2 presents the centrality measures of each node in the network linking BF and caregiving abilities. The findings demonstrate that the social relationships (BF dimension 4) exhibits the highest strength centrality, followed by the family relationships (BF dimension 2). Further, bootstrapped tests on the strength of each node reveal that the social relationships (BF dimension 4) has the highest strength centrality, showing statistically significant differences from all other dimensions. The family relationships (BF dimension 2) ranks second, demonstrating statistically significant differences with all dimensions except for personal growth and health behaviors. The stability coefficient CS = 0.596 (>0.5) indicates good stability of the strength centrality measures across the network nodes.

### 3.6 Bridge strength centrality index and identification of bridge nodes

**Figure 3:**
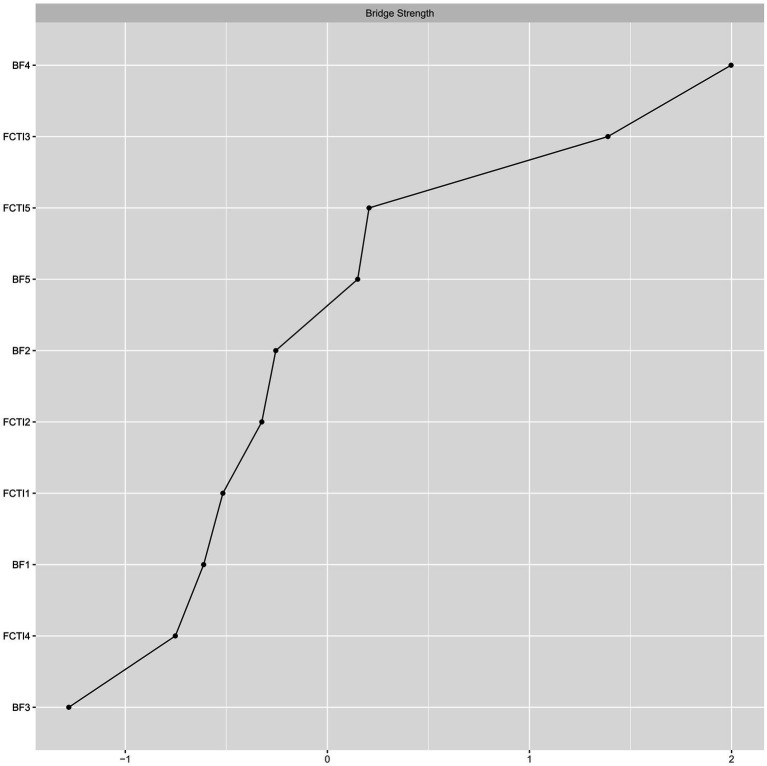
Bridge strength centrality index of network nodes.

Figure 3 depicts the bridge strength centrality of nodes in the overall network. The social relationships (BF dimension 4) emerges as the node with the highest bridging strength centrality, followed by the adjusting personal needs (FCTI dimension 5). Further conducting bootstrapped tests on the strength of each bridge node reveals that the social relationships (BF dimension 4) exhibits the highest strength, followed by the dimension of adjusting personal needs (FCTI dimension 5). However, there is no statistically significant difference between these two nodes. The stability coefficient CS = 0.438 (>0.25) indicates a reasonably stable measure of strength bridging centrality.

## 4 Discussion

### 4.1 Social relationship dimension is the most vital core node and bridge node in the network

The social relationships (BF dimension 4) assumes a central role as a core node within the entire network, functioning as a bridge node that connects the BF and the caregiving abilities. It demonstrates a close relationship with the personal growth (BF dimension 3), signifying its vital centrality in the network. Social relationships encompass individuals’ interpersonal connections with close relatives, colleagues, friends, and other socially relevant individuals. This entails the structural aspects of social relationships, including their size, scope, and connections, as well as the functional aspects, such as interactivity and cohesion among social relationship structures (Yang et al., 2022). The quality of social relationships indicates an individual’s perceived support from family and friends, and a favorable quality of social relationships is an essential precondition for individuals to access social support. Professor Bond (Becker et al., 2012) from Harvard University conducted in-depth research on social relationships, demonstrating that positive interpersonal relationships can ameliorate individuals’ negative cognitions and enhance their subjective well-being.

Studies have provided evidence (Pearce et al., 2023; Sabatini et al., 2024; Noguchi et al., 2020) that the quality of social relationships serves as an external resource available to individuals when dealing with stressful events and is closely associated with caregiving abilities and psychological well-being. Within the present study’s social relationships dimension, three items were included: ‘I have met some people who have become my friends,’ ‘It helped me feel cared for and supported by others,’ and ‘It helped me realize who my true friends are.’ These items indicate that positive social relationships play a constructive role for caregivers during their caregiving responsibilities. These relationships enable family caregivers to maintain an optimistic mood, adapt to their caregiving role more effectively, and enhance their confidence in the patient’s treatment. Furthermore, positive social relationships encourage caregivers to actively participate in the disease recovery process with the assistance of their family and friends, facilitating the reestablishment of intimate family relationships and social engagement (Sabatini et al., 2024). Therefore, the quality of social relationships holds noteworthy significance for patients and caregivers in the context of disease treatment and restoring health.

The social relationships dimension, acting as a significant node closely connected to other nodes, indicates that healthcare professionals can activate various nodes within the network, such as personal growth, through interventions targeted at this specific node. Such interventions have the potential to enhance both the overall caregiving abilities and levels of BF among family caregivers of patients with lung cancer. Throughout the caregiving process, caregivers demonstrate a strong desire to experience spiritual or material care and understanding from their relatives, friends, and the broader society. Encouraging caregivers to engage in communication and interaction with their family members and friends and express their inner concerns should be actively encouraged. Considering the joint employment of combined treatments, such as radiotherapy and chemotherapy, for lung cancer patients, healthcare providers can leverage the opportunity of patients returning for scheduled treatments to provide increased support and emotional comfort to caregivers, thereby encouraging them to adopt an optimistic and compassionate mindset towards their caregiving role. For caregivers who face challenges in physically distancing themselves from critically ill patients or those who exhibit introverted tendencies, healthcare professionals can consider offering psychological support and effectively utilizing social resources to assist in establishing and maintaining positive social relationships. Moreover, organizing support groups or ward activities and establishing caregiver communication groups can provide platforms for caregivers to share their experiences and emotions with others, alleviating their burden and providing emotional support.

### 4.2 Family relationship dimension is an important core node in the network

The family relationships (BF dimension 2) serves as a secondary core node in the network, indicating its potential influence on other nodes and emphasizing the critical role of optimizing family relationships throughout the caregiving process. Compared to the social relationships dimension, family relationships are considered a specific manifestation of social connections, providing a more accurate reflection of their significance for caregivers. Increasingly, research (Wu et al., 2023; Kishino et al., 2023) recognizes that treating illnesses relies not only on the individual strength of the patient but also on a family-centered care approach. Family-centered care considers the physiological, psychological, and social conditions of both the patient and family members, guiding active involvement in treatment plans, decision-making, and caregiving processes, ultimately enhancing disease management collectively.

A qualitative interview conducted by Liu et al. (2023) among advanced cancer patients and caregivers revealed that over 90% of participants emphasized the significance of the family as a warm sanctuary and a vital source of social support. The sense of belonging within the family motivated them to resist the disease and provide mutual companionship. Network analysis exploring the relationship between adolescent loneliness and family relationships (Heshmati et al., 2021) demonstrated a strong correlation, particularly highlighting that high-quality family relationships can alleviate feelings of loneliness. Additionally, the quality of family relationships does not necessarily depend on the quantity of relationships; a positive correlation does not exist between the quantity and quality of family relationships. Research (Ren et al., 2019) suggests a close association between positive emotions and positive family relationships. If negative emotions permeate within family members, the household may be enveloped in a hostile and gloomy atmosphere. Over time, the family dynamic erodes, rendering them unable to confront external stressors collectively. Tense family relationships not only fail to benefit caregivers but also impede the improvement of the patient’s condition. Lindeza et al. (2020), in their systematic review of caregivers of dementia patients, highlighted that positive family relationships not only encourage primary caregivers to sustain caregiving but also motivate other family members to participate in caregiving. This further enhances family members’ awareness of health and cohesion while improving their caregiving abilities, thereby establishing a robust family support system for the patients. Koehly et al. (2015) emphasized the importance of considering the family as a focal point in caregiving network analysis, suggesting a comprehensive exploration of various family characteristics, including interaction patterns, communication modes, support systems, and interdependence among family members, to gain a holistic understanding of the impact of the family on caregivers.

Therefore, this study advocates for leveraging the role of healthy family relationships in offering emotional and informational support to lung cancer patients. Healthcare professionals should not solely focus on the number of family members but should also consider additional family characteristics such as intimacy among relatives, number of relatives, and household size (Koehly et al., 2015). Through effective communication with family members, healthcare professionals can assess familial harmony, mutual support, understanding, and collaboration. It is crucial to encourage caregivers to approach challenges with a positive mindset, starting with fostering a harmonious family atmosphere, providing adequate patient care, and enhancing the family’s resilience and capacity to cope with caregiving difficulties.

### 4.3 Adjusting personal needs dimension is an essential bridging node in the network

The adjusting personal needs (FCTI dimension 5) serves as a crucial bridging node connecting the BF and caregiving abilities, highlighting the significance of effectively managing caregivers’ personal emotions in enhancing BF and caregiving abilities among family caregivers of patients with lung cancer. Firstly, caregivers’ personal emotions share similarities with BF as they both represent internalized expressions of individuals’ psychological states. A cross-sectional study conducted by An et al. (2023) on caregivers of children with Mediterranean anemia found that emotion regulation ranked second among various dimensions, indicating its importance as a manifestation of caregivers’ caregiving abilities. Furthermore, research (Huang et al., 2019) suggests that effective emotion regulation is a critical issue in improving caregivers’ caregiving abilities. Within this dimension, “eliminating uncertainty about caregiving skills” emerges as a significant item, aligning with previous studies (Yue et al., 2022) that highlight caregiving skills as a core factor influencing BF and caregiving abilities. Faced with adverse reactions and nutritional needs resulting from lung cancer, family caregivers encounter a range of caregiving challenges, such as tube blockage or displacement of nasal or gastric tubes. Caregivers with systematic training can effectively manage unexpected events and changes in the patient’s condition, leading to lower levels of disease benefit finding. Therefore, focusing on caregiving skills serves as a practical approach to enhance caregivers’ control over their personal emotions, ultimately influencing the levels of BF and caregiving abilities.

Hence, this study suggests that healthcare professionals can strengthen emotional support for family caregivers, encouraging them to share their negative emotions with friends and family to alleviate inner distress. Additionally, demonstrating correct caregiving techniques and procedures through recorded instructional videos, scenario simulations, and demonstrations (Kapanee et al., 2022) can help caregivers gain a visual understanding of caregiving, enabling them to grasp caregiving skills better, enhance their caregiving confidence and ultimately perceive the benefits of caregiving.

### 4.4 Limitations

Future research endeavors should focus on expanding the sample size to enhance the statistical power and generalizability of the findings. Furthermore, it is crucial to thoroughly consider caregiving characteristics, such as the number of caregivers and the duration of caregiving to obtain a comprehensive understanding of the caregiving process. Longitudinal studies employing dynamic network analysis techniques can provide valuable insights into the evolving nature of caregiving relationships over time. By examining changes in network dynamics and investigating the reciprocal influences among caregivers and other relevant factors, these studies can offer a more nuanced understanding of the complex dynamics within caregiving networks.

## 5 Conclusion

This study aimed to construct a network to investigate the relationship between disease benefit finding and caregiving abilities in lung cancer caregivers. The findings provide valuable insights for healthcare professionals to identify new intervention targets in order to enhance the caregiving abilities and levels of BF among informal lung cancer caregivers. Interventions targeting improvement in social relationships, family dynamics, and emotion regulation could be effective in enhancing the caregiving abilities and promoting the experience of benefit finding among caregivers. These findings have important implications for developing tailored interventions and support programs that address the specific needs and challenges faced by lung cancer caregivers, ultimately enhancing their overall well-being and caregiving outcomes.

## Data Availability

The raw data supporting the conclusions of this article will be made available by the authors, without undue reservation.
